# Hypertension and headache: a coincidence without any real association

**DOI:** 10.1590/S1516-31802003000500001

**Published:** 2003-09-01

An association between hypertension and headache was first proposed by Janeway in 1913.^1^ A lot of studies were done subsequently, and conflicting results have emerged from investigations into the relationship between headache and arterial blood pressure.

Bulpitt et al. observed that 31% of patients with untreated, severe hypertension complained of headache, compared with 15% of treated hypertensive patients and controls without hypertension.^2^ Cooper et al., in a sample of 11,710 hypertensive patients, reported that headache was a common symptom related to high blood pressure levels.^3^ Rasmussen, in a Swedish population-based study, observed that women with migraine showed significantly higher diastolic blood pressure than those without migraine.^4^ In contrast, in another population-based study, Walters found no differences in systolic and diastolic blood pressure levels when individuals with headache were compared with individuals who had not had a headache in the previous year.^5^

The most interesting study was done by Stewart in 1953. In this seminal paper, 200 consecutive patients with hypertension were studied, with 96 of them aware of their blood pressure status. Among the people aware of their hypertensive status, 71 (74%) complained of headache. In contrast, among the 104 people who were not aware of their blood pressure status, only 17 (16%) complained of headache. Stewart concluded that once people become aware of their hypertension diagnosis, the frequency of reported headache increases.^6^ Using data from the 1960–62 United States Health Examination Survey of Adults, Weiss observed no differences in the occur-rence of headache among 6,672 normotensive and hypertensive people who were not aware of their blood pressure status.^7^ In all these studies, blood pressure was measured in an office setting using a standardized technique.

In a Brazilian study, Benseñor et al., using 24-hour ambulatory blood pressure monitoring, did not demonstrate any difference in blood pressure levels between women with and without chronic daily headache.^8^ More recently, Hansson et al.^9^ concluded that the incidence of headache can be reduced by antihypertensive treatment, in a study using data from seven randomized, double-blind, placebo-controlled trials. These trials included 2,673 patients with mild-to-moderate hypertension to whom once-daily treatment with irbesartan (n = 1987) or placebo (n = 686) was administered. Hansson et al. also stated that in the Hypertension Optimal Treatment Study (HOT Study), intensive blood pressure-lowering control improved quality of life and significantly reduced the frequency of migraine headaches. However, there are now many studies questioning the role of angiotensin II receptor antagonist in migraine prophylaxis. In the HOT Study, 50% of patients were also randomized to aspirin, another drug used for migraine prophylaxis. Many patients are also using beta-blockers, another class of drugs for migraine prophylaxis.^10^

In another Brazilian study, Wiehe et al. evaluated the association between headache and hypertension in a sample of 1,174 individuals aged over 17 years, representative of inhabitants of Porto Alegre, Brazil. Headache over their lifetimes or in the last year, defined as episodic and chronic tension-type headache, was not associated with hypertension. Individuals with optimal or normal blood pressure (Sixth Joint National Committee criteria) complained of migraine more frequently than the participants with high-normal blood pressure or hypertension.^11^

Enough data are now available to conclude that headache and hypertension are not really associated. They are common complaints in emergency rooms and many people may complain of both without any evidence of association. The use of the International Headache Society (IHS) criteria to classify headaches allows the physician in the emergency room to make a correct diagnosis of the headache complaint, which is frequently migraine, and thus prescribe the correct treatment. Therefore, if a headache patient comes to the emergency room, doctors should first use the IHS criteria to classify his or her headache and, following this, if indicated, the blood pressure levels should be measured. It must be remembered that pain or the simple fact of being present in an emergency room can raise blood pressure levels.
